# Incidence and mortality rates of varicella among end stage renal disease (ESRD) patients in Singapore General Hospital, a 12-year review

**DOI:** 10.1186/s12879-018-3023-y

**Published:** 2018-03-07

**Authors:** Chong Yau Ong, Sher Guan Low, Farhad Fakhrudin Vasanwala, Stephanie, MC Fook-Chong, Manish Kaushik, Lian Leng Low

**Affiliations:** 1 Department of Family Medicine, Sengkang Health, New Office Building 20A, 378 Alexandra Road, Singapore, 159964 Singapore; 2 Health Services Research Unit, Division of Medicine, Singapore General Hospital, Singapore, Singapore; 3 Department of Renal Medicine, Singapore General Hospital, Singapore, Singapore; 4 Department of Family Medicine and Continuing Care, Singapore General Hospital, Singapore, Singapore; 5 SingHealth Duke-NUS Family Medicine Academic Clinical Programme, Singapore, Singapore

**Keywords:** Varicella, Chickenpox, End stage renal failure, End stage renal disease, Varicella vaccine

## Abstract

**Background:**

End stage renal disease (ESRD)/ end stage renal failure (ESRF) is on the rise globally and especially in Singapore. Varicella or chickenpox is not uncommon among adults especially ESRD/ESRF patients. It has been reported to cause complications and even death among immunocompetent adults.

**Methods:**

A retrospective data collection on patients with varicella infection and ESRD in Singapore General Hospital (SGH) from the year 2005 to 2016 was performed. Continuous data and categorical data were summarized as median (range) and count (%) respectively. The association of health care utilization (total length of hospital stay, readmission related to varicella, intensive care unit admission) and mortality with complication due to varicella were tested using chi-square and Mann-Whitney test for categorical and continuous outcomes respectively.

**Results:**

Sixty-six patients with ESRD developed varicella during the study period (2005–2016). The case incidence rates for varicella among ESRD ranges from 97 to 267 per 100,000 populations with ESRD yearly. There were 9 deaths (13.6%). Mortality was higher among the ESRD patients with one or more varicella complications compared to patients without complications ((25% vs 7.1%, 95% CI for difference: − 1.1%, 36.9%, *p* = .063). Likewise, utilisation of intensive or high dependency units were higher among patients with complications compared to those without (20.8% vs 2.4%, 95% CI for difference: 1.6%, 35.3%, *p* = .012). Length of stay was twice as long in the group with complications compared to patients without (median (IQR) days: 14 (8, 21) vs 7 (5, 14), *p* = .065), although it did not reach statistical significance.

**Conclusions:**

Varicella is associated with high morbidity and significant mortality rate in ESRD patients. Varicella vaccination is recommended for seronegative ESRD patients.

## Background

Patients suffering from chronic kidney disease (CKD) and end stage renal disease (ESRD) / end stage renal failure (ESRF) (both ESRF and ESRD are henceforth referred to as ESRD) are rising worldwide. Incidence rates of ESRD/ESRF is also rising for many developing countries [1]. It was estimated there were 2.6 million people on dialysis in 2010; 93% from high or upper middle-income countries [2]. Worldwide use of renal replacement therapy (RRT) projected to more than double by 2030, with the most rapid increase in Asia [3]. ESRD patients have impaired immune system and are susceptible to serious infections [4]. Patients on haemodialysis (HD) have 14–16 fold increased risk of mortality from pulmonary infections as compared with general population [5].

Primary varicella or varicella (henceforth referred to as varicella) which is more commonly known as chickenpox is an acute infectious disease that is caused by varicella zoster virus (VZV), an alpha herpes virus belonging to the Herpesviridae family. Varicella is highly contagious, with secondary household attack rate of over 90% [6]. Varicella zoster virus is transmitted mostly through airborne, and also by direct contact with vesicular fluids. Usually the course of the disease is benign; however it can lead to severe complications and mortality both in immunocompromised and immunocompetent patients as well. Clinical illness is mild for all immunocompetent hosts although disease severity increases with age. Adults have 10–20 fold increase in rates of varicella pneumonia and 3 to 17 fold higher rates of hospitalization for varicella or related complications [7]. The list of complications from varicella include pneumonia, pneumonitis, acute obstructive respiratory disease, encephalitis, meningitis, neutropenia, thrombocytopenia, Henoch Schonlein purpura, synovitis, Reye’s syndrome, just to name a few. It can also result in secondary bacterial infections that manifest as sepsis, cellulitis, impetigo, abscesses, necrotizing fasciitis, and toxic skin syndrome. Reactivation of dormant varicella-zoster virus within dorsal root ganglia results in herpes zoster (shingles) or less commonly secondary varicella. This can manifests decades after the initial exposure [8].

In Singapore, varicella considered an endemic disease. There were a total of 3987 attendances in polyclinics (health clinics) for chickenpox in 2014 and more than one third of the attendances (1378) were adults over 20 years old [9]. It was reported that the annual incidence rate per 100,000 populations in Singapore was 636.1 in 1989 and 1355.7 in 1996. The annual incidence rates per 100,000 population was between 371 and 665.7 during the period 2002–2007. It is possible that the introduction of varicella vaccine to Singapore in 1996 had contributed to the declining trend [9]. From 1992 to 2011 in Singapore, there were 46 deaths due to varicella (chickenpox), mainly among adults and elderly.

Evidence published to date suggests that varicella vaccination are effective and safe in ESRD and patients on renal replacement therapy [10–13]. Common adverse effects include redness, pain and swelling at the injection site, fever, headache, myalgia, nausea and itching. There have been several guidelines published internationally recommending varicella vaccination for ESRD or even CKD [14–17]. The Advisory Committee Immunization Practices (ACIP) recommends that all children and adults without evidence of immunity receive two doses of the vaccine; those who received only one dose of vaccine should receive a second dose [14].

However in Asia region, varicella vaccination in ESRD patients is not widely practiced due to lack of national or regional consensus guidelines. There have been no recommendations made by the local health authorities in Singapore on the role of varicella vaccination among those with ESRD. To our best knowledge, there are no published local data on the risk of VZV infection among ESRD patients. [18]. Therefore this study was done to measure the case incidence rates, mortality and morbidity rates of varicella among ESRD patients in our local context.

## Methods

A retrospective data collection on patients with varicella infection and ESRD in Singapore General Hospital (SGH) from the year 2005 to 2016 was performed. Singapore is an island city-state off South-East Asia with dense population of 5.6 million (in 2016) within the 719km^2^ (277.6 sq. mi.). It has one of the most rapidly ageing population in Asia with increasing patients suffering from chronic diseases. Health care expenditure in Singapore projected to triple from S$4 billion in 2011 to S$12 billion in 2020 with 10,000 additional hospital beds required [19]. SGH is the first and largest hospital in Singapore. SGH treats about half to two-thirds of patients with ESRD and CKD in Singapore [4].

The study period was defined from 1st January 2005 to 31st December 2016. The ethics approval was obtained from SingHealth Centralised Institutional Research Board (CIRB) (Reference: 2016/2780).

The inclusion criteria were patients with ESRD or ESRF or CKD Stage 5 – estimated Glomerular Filtration Rate (GFR) < 15 mL/min (regardless if the patient was on renal replacement therapy or on conservative management of patient’ kidney failure) that contracted varicella infection during the study period of 2005–2016. Varicella infections included varicella without complication and complications such as pneumonia, varicella pneumonitis, varicella encephalitis, varicella meningitis, and other complications. Exclusion criteria were patients with herpes zoster (secondary varicella infection), varicella without underlying ESRD/ESRF/CKD Stage 5 and patients with ESRD/ESRF/CKD Stage 5 but who did not develop varicella.

Electronic medical records were searched with the both International Classification of Diseases, ICD- 9 and ICD-10 codes [20, 21]. ICD-9 codes for ESRD/ESRF/CKD Stage 5 included these starting with 403.01, 403.11, 403.91, 404.02, 404.03, 04.12, 404.13, 404.92, 404.93, 581.x, 582.x, 583.0–583.7, 585.x, 586.x, 588.0, V42.0, V45.1, and V56.x. For ICD-10, we included codes starting with I12.0, I13.1, N18.5, N18.6, and Z99.2. As for varicella, codes starting with 052.x were used in ICD-9 and codes starting with B01.x were used in ICD-10.

Data was extracted by the department of information technology, SGH. Data was checked manually by three authors independently to ensure accuracy of the extraction. Initial data that does not fulfil the inclusion criteria or that fulfilled the exclusion criteria was excluded.

Data on demographics (age, sex, gender, ethnic group, and occupation), cardiovascular comorbidities (hypertension, diabetes, dyslipidaemia, ischaemic heart disease, coronary artery disease, peripheral vascular disease), and details of renal disease (years being diagnosed with ESRD/ESRF, whether patient on renal replacement therapy, and modality of renal replacement therapy) were retrieved. Presence of previous varicella exposure was determined by serology test of positive varicella immunoglobulin G (IgG) during the presentation of varicella infection. Immunoglobulin M (IgM) was tested using Indirect Fluorescent Antibody (IFA) (IFA, Hemagen Diagnostic Incorporation, United States of America, USA) and IgG was tested using Enzyme Immunoassay (EIA) (EIA, HUMAN Diagnostics, Germany). Number of end organ complications caused by varicella infection, length of hospital stay (admission), length of intensive care or high dependency units (ICU/HDU) stay, and readmissions were captured as a measure of burden of disease.

All statistical analysis was conducted using the International Business Machine Corporation (IBM) Statistical Package for Social Sciences (SPSS) version 24.0. Continuous data and categorical data were summarized as median (range) and count (%) respectively. The association of health care utilization (total length of hospital stay, readmission related to varicella, Intensive Care Unit (ICU) admission) and mortality with complication due to varicella were tested using chi-square and Mann-Whitney test for categorical and continuous outcomes respectively. Complication due to varicella was defined as 1 or more of the following complications: varicella encephalitis, varicella meningitis, varicella pneumonitis, hepatitis, varicella keratitis, and other varicella complications.

## Results

During the 12 year observation period (2005–2016), a total of 66 patients with ESRD were admitted to SGH for primary varicella infection. Patients had a median age of 53 years and were predominantly male (56.1%), Table 1. Three quarter of the patients were Chinese (77.3%). Most of the patients had existing cardiovascular co-morbidities: hypertension (87.9%), dyslipidaemia (66.7%), diabetes (30.3%), ischaemic heart disease (25.8%), and coronary artery disease (22.7%).

**Table 1:** Patient, clinical characteristics and complications

Characteristics	Summary statistics; count (%) or median (range) (*N* = 66)
Age (in years)	53 (19, 89)
Gender	
Female	29 (43.9%)
Male	37 (56.1%)
Race	
Chinese	51 (77.3%)
Malay	12 (18.2%)
Indian	1 (1.5%)
Others	2 (3.0%)
Cardiovascular risk factors	
Hypertension	58 (87.9%)
Diabetes	20 (30.3%)
Dyslipidaemia	44 (66.7%)
Ischaemic heart disease	17 (25.8%)
Coronary artery disease	15 (22.7%)
Number of years being ESRD before onset of varicella. (available data *n* = 37)	6 (0–19)
Dialysis (available data *n* = 59)	
Yes	42 (71.1%)
No	17 (28.9%)
Renal transplantation	
Yes	23 (34.8%)
No	43 (65.2%)
Status post renal transplantation (available data *n* = 23)	
Functioning graft kidney	14 (60.8%)
Failed graft	9 (39.2%)
Previous immunity (available data n = 19)	
Yes (seropositive to VZV IgG)	11 (57.9%)
No (seronegative to VZV IgG)	8 (42.1%)
Length of stay (days)	10 (2, 2555)
ICU/HD stay	
Yes	6 (9.1%)
No	60 (90.9%)
Death	
Yes	9 (13.6%)
No	57 (86.4%)
Readmissions (within 30 days)	
Yes	55 (83.3%)
No	11 (16.7%)

The median duration between diagnosis of ESRD and documented admission for primary varicella infection was 6 years. One patient developed varicella 19 years after being diagnosed with ESRD. Only 19 patients who developed varicella have documentation on prior VZV antibody status (i.e. VZV IgG positive or negative) on medical records during the index admission for varicella infection. Among the 19 patients, 8 (42.1%) were found to be seronegative (negative IgG- class antibodies), Table 1.

More than two-thirds (71%) had dialysis as the mode of renal replacement therapy, Table 1. 23 of the patients had renal transplantation (34.8%). Among the renal transplant recipients, 60.8% still has functioning kidney at the time of study. Among 23 renal transplanted recipients, 2 died (8.7%) during the 12-year review.

The case incidence rates for varicella among ESRD were low; ranging from 97 to 267 per 100,000 population per year. Mortality rate among patients with primary varicella on the background of ESRD was 13.6%. Among the nine patients that died of varicella, two were renal transplant recipients, four were haemodialysis patients, and the remainder three were not on any renal replacement therapy. Two demised patients had varicella encephalitis and pneumonia, two with varicella encephalitis, and two with varicella pneumonia. During the study period, there were 81 reported deaths from varicella without ESRD. 11.1% of the patients who died of varicella had an underlying ESRD.

**Fig. 1:**
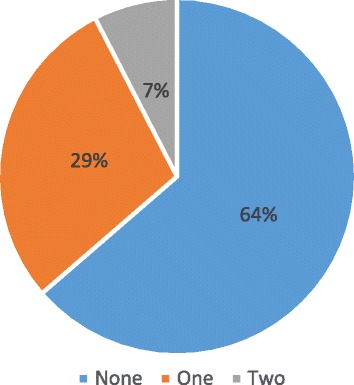
Number of organ complications occurring in patients with ESRD during varicella infection

As for morbidities, 24 (36.4%) patients developed at least one complication from primary varicella (see Fig. 1). These included encephalitis, pneumonia or pneumonitis, and meningitis. 9% of the patients had admission either in the intensive care unit or high dependency unit. 83.3% of the patients had readmission within 30 days from varicella and non-varicella related illnesses. Median length of stay was 10 days. Length of stay was twice as long in the group with complications compared to patients without (Table 2).

**Table 2:** Association of varicella complication with outcomes. Table 2 shows that death (25% vs 7.1%, *p* = .042) and intensive care unit (ICU) or high dependency (HDU) admissions (20.8% vs 2.4%, *p* = .012) and, total length of hospital stay (median days: 14 vs 7, *p* = 0.065) were higher among the ESRD patients with 1 or more complications arising from varicella infection compared to patients with no complication

Outcome	Complication (Cx) from varicella infection		Difference in outcome (Cx group - No Cx group)	*p*-value
	No (*N* = 42)	Yes (*N* = 24)		
ICU/ High dependency	1 (2.4%)	5 (20.8%)	18.4% (1.6%, 35.3%)	0.012
Death from varicella admission	3 (7.1%)	6 (25%)	17.9% (−1.1%, 36.9%)*	0.042 (0.063 by Fisher’s Exact test)
Total length of stay in hospital (days), median, [IQR]	7 [5,14]	14 [8, 21]	ND	0.065
Readmission related to varicella admission, within 30 days	14 (33.3%)	6 (25%)	−8.3% (−30.8%, 14.1%)	0.479

IQR; Interquartile Range

*95% CI was computed by exact test

ND; mean difference (95%CI) not done as median is reported instead of mean

## Discussion

We found that primary varicella infection resulted in a significant morbidity and mortality among patients with ESRD. Overall mortality rate among this inpatient group of patients was 13.6%. Deaths were higher among patients with one or more system involvement compared to those who had none. As for morbidity, the utilisation of higher degree of care support was evident given that those with one or more organ system involvement had more admissions into the intensive care unit (ICU) and high dependency unit (HDU). Patients with one or more organ system involvement stayed longer in hospital compared to those without complications although this was not significant statistically.

The demographics of our patients with ESRD and varicella was similar to the national demographics [4]. Slightly more than half of the patients were male which is comparable to national data. The predominant race in this group of patients is the Chinese which is comparable to national data; 77.3% vs 65.3–72% [4].

Our mortality rate obtained was consistent with Fehr’s findings (2002) where mortality rates of varicella among renal allograft recipients were 22% after 1990 [22]. Prior to the introduction of acyclovir the overall mortality rates was 34% [22]. One ten year retrospective analysis of renal allograft patients showed that varicella associated with an increased mortality of 13.4% [23]. To our knowledge and review of literature, there were no reports on mortality rates among patients with ESRD on dialysis and ESRD not on renal replacement therapy for direct comparison. Most varicella patients died of multiorgan failure (from hepatitis, encephalitis, pneumonitis, and disseminated intravascular coagulation) [24–27], and respiratory failure [28].

There was no similar data on case incidence of varicella among ESRD patients obtained from literature. Case incidence of varicella among renal allografts recipients has been reported at 0.84 to 1% [24, 25].

Although the available data for previous immunity (VZV IgG) among the infected patients was small (*n* = 19), VZV seronegative of 42% is not much different from results from literature. Rodriguez reported eight patients with varicella infection; of whom four (50%) were tested negative for VZV IgG [25]. Similarly, Abad et al. reported a baseline serology of 32 cases with disseminated varicella among renal transplant patients; of which 59.4% were seronegative [29].

At present we have no data on the prevalence of VZV seropositivity among non-varicella infected ESRD patients. Published reports revealed that among candidates or recipients of renal transplant (without varicella); the prevalence of seronegative patients were much lower at 2.1 to 9.8% [12, 13]. The 2010 National Health Survey using residual blood samples from healthy volunteered adults (comorbidities unknown) showed that seroprevalance of varicella antibodies among adults (17–79 years old) in Singapore was around 88% [29]. In view that ESRD patients are at higher risk due to their immunocompromised state, we should aim for a seroprevalance (positive IgG) among ESRD patients that is similar to the 88% among healthy adult community in Singapore [30] or even more in view that their immunocompromised state.

### Limitations and future plans

The main limitation of this study was the lack of complete documented data from retrospective study as it is wholly dependent on the thoroughness of the discharge summary which is usually done by the junior doctors. These information included the occupation of the patients, the underlying cause leading to ESRD/ESRF, previous immunity to varicella. The degree of complications and organ system involvement could also be underreported if it was not dedicatedly reported in the diagnosis column by the managing team. We minimised this limitation by a thorough check of both the electronic medical records and discharge codes.

The second limitation is that the data collected was from a single centre study. However, the Singapore General Hospital treats the majority of renal patients in Singapore (up to 65.4%) and our patient demographics was similar to the national demographics for ESRD patients. In future studies, pooling of data from other major centres in Singapore looking after renal patients would give a more comprehensive information of varicella infection among ESRD patients in Singapore.

Cost analysis studies is yet to be done to accurately quantify the full extent of morbidity among this group of patients. Despite having chronic disease of ESRD, most of the patients were still working adults. Admission from varicella definitely indirectly incur major impacts such as absences from work, loss of income, and effect on other family members having need to take time out to take care of patients being hospitalised.

### Implications for clinical practice

ESRD patients are immunocompromised, and renal transplantation recipients require immunotherapy agents. Therefore patients with ESRD are highly susceptible for communicable disease. It is not uncommon for the patients to contract primary varicella infection. Once infected, varicella has been proven to be highly lethal disease; more than one in eight will succumbed to fatality from varicella infection. Whilst in other more developed countries, varicella vaccination has been introduced as national immunisation schedule leading increased immunity, this is not so with Singapore. We therefore recommend that screening of previous immunity to be carried out among patients with ESRD, regardless of their status of renal replacement therapy be it for renal transplant or not. Those who have no previous immunity to varicella should be given two doses of live attenuated varicella vaccine.

Family physicians in Singapore treat the largest pool of patients in the country. Advocating vaccinations for example pneumococcal, influenza, and human papillomavirus to the group of patients has always being in the sphere of primary care duties of family physicians. This should be extended to include varicella vaccination to the end stage renal disease patients in view of the significant morbidity and mortality of varicella infection to this group of patients.

## Conclusions

In our study, we found that ESRD patients had significant morbidity and mortality associated with primary varicella infection. ESRD patients should be screened for immunity to varicella and those without immunity to varicella considered for varicella vaccination, in accordance to well-established international guidelines.

## Abbreviations

CI: confidence interval
CKD: chronic kidney disease
EIA: enzyme immunoassay
ESRD: end stage renal disease
ESRF: end stage renal failure
GFR: glomerular filtration rate
HD: haemodialysis
HDU: high dependency unit
IBM: International Business Machine Corporation
ICD: International Classification of Diseases
ICU: intensive care unit
IFA: indirect fluorescent antibody
IgG: immunoglobulin G
IgM: immunoglobulin M
IQR: interquartile range
km^2^: kilometre square
min: minute
ml: millilitre
n: number
p: *p*-value
RRT: replacement therapy
SGH: Singapore General Hospital
USA: United States of America
vs: versus
VZV: varicella zoster virus
